# The roles of Bcl-x_L _in modulating apoptosis during development of *Xenopus laevis*

**DOI:** 10.1186/1471-213X-5-20

**Published:** 2005-09-26

**Authors:** Jillian Johnston, Robert Chan, Maria Calderon-Segura, Sarah McFarlane, Leon W Browder

**Affiliations:** 1 Genes and Development Research Group, Department of Biochemistry & Molecular Biology, University of Calgary, Calgary, Alberta, Canada, T2N 4N1; 2 Genes and Development Research Group, Department of Cell Biology & Anatomy, University of Calgary, Calgary, Alberta, Canada, T2N 4N1; 3 Universidad Nacional Autónoma de México, Centro de Ciencias de la, Atmósfera, Laboratorio de Citogenética Ambiental, Circuito Exterior S/N, Col. Coyoacán, Ciudad Universitaria, C.P.0451, México, Distrito Federal

## Abstract

**Background:**

Apoptosis is a common and essential aspect of development. It is particularly prevalent in the central nervous system and during remodelling processes such as formation of the digits and in amphibian metamorphosis. Apoptosis, which is dependent upon a balance between pro- and anti-apoptotic factors, also enables the embryo to rid itself of cells damaged by gamma irradiation. In this study, the roles of the anti-apoptotic factor Bcl-x_L _in protecting cells from apoptosis were examined in *Xenopus laevis *embryos using transgenesis to overexpress the *XR11 *gene, which encodes Bcl-x_L_. The effects on developmental, thyroid hormone-induced and γ-radiation-induced apoptosis in embryos were examined in these transgenic animals.

**Results:**

Apoptosis was abrogated in *XR11 *transgenic embryos. However, the transgene did not prevent the apoptotic response of tadpoles to thyroid hormone during metamorphosis. Post-metamorphic *XR11 *frogs were reared to sexual maturity, thus allowing us to produce second-generation embryos and enabling us to distinguish between the maternal and zygotic contributions of Bcl-x_L _to the γ-radiation apoptotic response. Wild-type embryos irradiated before the mid-blastula transition (MBT) underwent normal cell division until reaching the MBT, after which they underwent massive, catastrophic apoptosis. Over-expression of Bcl-x_L _derived from *XR11 *females, but not males, provided partial protection from apoptosis. Maternal expression of XR11 was also sufficient to abrogate apoptosis triggered by post-MBT γ-radiation. Tolerance to post-MBT γ-radiation from zygotically-derived XR11 was acquired gradually after the MBT in spite of abundant XR11 protein synthesis.

**Conclusion:**

Our data suggest that Bcl-x_L _is an effective counterbalance to proapoptotic factors during embryonic development but has no apparent effect on the thyroid hormone-induced apoptosis that occurs during metamorphosis. Furthermore, post-MBT apoptosis triggered by irradiation before the MBT could only be restrained by maternal expression of *Bcl-x*_*L*_. Although maternal expression of XR11 was sufficient to abrogate apoptosis triggered by post-MBT γ-radiation, radiation tolerance from zygotically-derived XR11 was acquired gradually, indicating that synthesis of XR11 protein is not sufficient to prevent apoptosis. Thus, repression of radiation-induced apoptosis by overexpression of Bcl-x_L _during embryonic development depends upon the timing of its expression and post-translational events that enable the protein to become effective.

## Background

Cell death is an essential and integral aspect of embryonic development (for reviews, see [1-3]). The genome is programmed to eliminate certain cells or groups of cells at particular times during development by a process called "programmed cell death" (PCD) [4-6]. Well-known examples of PCD during vertebrate development include interdigital cell death and regression of the tail and gills during amphibian metamorphosis [3]. Considerable PCD also occurs in the central nervous system (CNS; for review, see [7]), where as much as 85% of some neuronal populations may be lost by PCD during development [8-11]. One form of PCD is apoptosis, which is characterized by membrane blebbing, nuclear and cytoplasmic shrinkage, chromatin condensation and DNA fragmentation [12]. The detection of DNA fragmentation in dying cells utilizing the TUNEL (TdT-mediated dUTP nick end labeling) assay provides the basis for a sensitive and specific *in situ *measure of apoptosis.

The frog *Xenopus laevis *provides an excellent model system for studying apoptosis during embryonic development. The TUNEL assay has been used on whole-mounts and tissue sections of *Xenopus *embryos to monitor spontaneous developmental apoptosis [13,14]. Apoptosis also provides the means for the embryo to rid itself of cells damaged by DNA-damaging agents, such as gamma radiation [15]. Gamma radiation during embryonic development is a threat to the viability of the embryo and to its potential to develop into a healthy, functional adult organism. Embryos have two alternative mechanisms to prevent developmental abnormalities caused by irradiation: elimination of damaged cells through apoptosis or repair of the damaged DNA. *Xenopus *embryos undergo a dramatic transition in their response to ionizing radiation coincident with the onset of gastrulation. Embryos irradiated before gastrulation continue to cleave normally and undergo an abrupt, catastrophic, comprehensive and synchronous apoptosis of DNA-damaged cells after the mid-blastula transition (MBT) using a maternally-derived mechanism that does not require transcription [15-20]. In contrast, cells in embryos irradiated after gastrulation reportedly do not undergo apoptosis [17,19,20].

The induction of apoptosis is subject to both pro- and anti-apoptotic cellular factors and is executed by cysteine proteases called caspases after their activation from the proenzyme state. The activation of caspases can be triggered by cytochrome c release from the intermembrane spaces of mitochondria through mitochondrial outer membrane permeabilization [21-24]. Release of cytochrome c, in turn, is regulated by members of the Bcl-2 family (for reviews, see [23,25,26]). Members of the Bcl-2 family serve a key regulatory role in apoptosis, acting as either pro- or anti-apoptotic factors. Thus, the relative levels of members with opposing functions can determine whether cells live or die [27] (for review, see [26]). One of the anti-apoptotic members of the Bcl-2 family is Bcl-x_L_, which functions to inhibit apoptosis in situations in which Bcl-2 does not suffice [28]. For example, *bcl-2 *knockout mice are viable as embryos [29,30] whereas *bcl-x*_*L *_knockout mice die as embryos that exhibit massive cell death in the nervous and hematopoietic systems [31]. Over-expression studies conducted both *in vitro *and *in vivo *in mice indicate that Bcl-x_L _is also *sufficient *to promote survival of embryonic neurons [32-34]. Similar results have been obtained with chick embryonic neurons *in vitro *[35]. Members of the Bcl family also modulate the apoptogenic effects of DNA-damaging agents. For example, lymphoid cells from *bcl-2 *null mice are hypersensitive to irradiation *in vitro *[29,30]. Conversely, over-expression of *bcl-2 *reduces the rate of apoptosis of γ-irradiated hematopoietic cells both *in vitro *and *in vivo *[36,37].

Cruz-Reyes and Tata [38]cloned two *bcl-*related genes in *Xenopus laevis*, which they designated as *XR1 *and *XR11*. XR11 [GenBank:X82461], which most closely resembles Bcl-x_L_, is expressed as early as stage 12 (gastrula) and continues to be expressed throughout embryonic, larval and adult life. Over-expression of XR11 in Rat-1 fibroblasts protected the cells from apoptosis, thus demonstrating that XR11 is an anti-apoptotic member of the Bcl family. Misexpression of human Bcl-2 or XR11 in *Xenopus *embryos has been shown to impede developmental- or chemically-induced apoptosis, respectively, during early *Xenopus laevis *embryonic development [17,39]. The transgenesis technique developed by Kroll and Amaya [40] provides the means to study the effects of genes on later developmental events and to examine the effects of the transgenes on subsequent generations. For example, Coen *et al. *[41] employed a neuron-specific promoter driving XR11 over-expression to demonstrate selective protection of Rohon-Beard cells during metamorphosis. In this report, we have used a constitutive promoter to examine the effects of generalized XR11 over-expression throughout *Xenopus *development and to examine the respective roles of maternal and zygotic XR11 in protection against γ-radiation-induced apoptosis during development.

## Results

### Over-expression of XR11 dramatically reduced spontaneous developmental apoptosis in embryos

**Figure 1:**
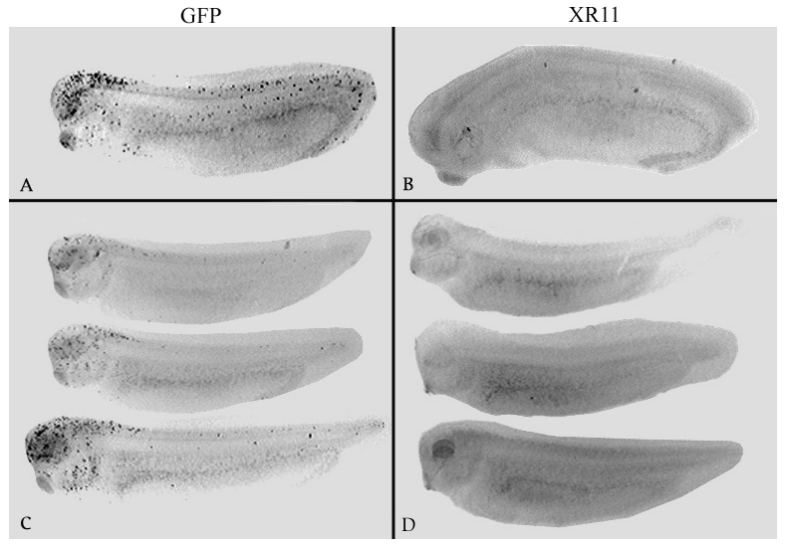
**TUNEL assays on whole-mount tailbud-stage embryos at stages 28(*A*, *B*) and 33/34 (*C*, *D*)**. A, C: GFP transgenic embryos B, D: XR11 transgenic embryos.

**Figure 2:**
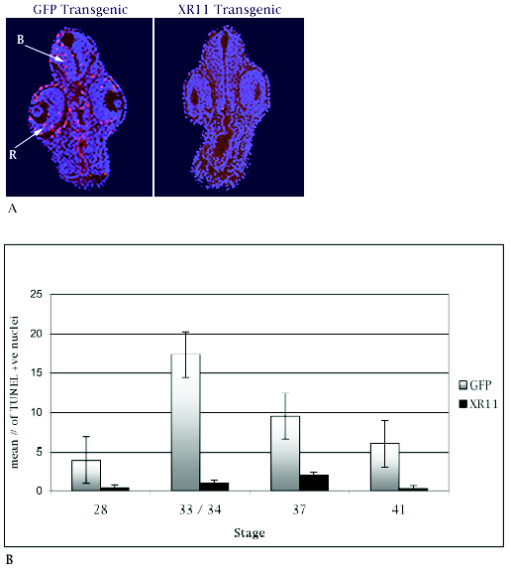
**Comparisons of TUNEL-positive nuclei between GFP and XR11 transgenic embryos during embryonic development**. ***A***. Cross-sections of embryos showing the distribution of TUNEL-positive nuclei at the level of the eye at stage 33. DAPI (blue) and TUNEL (red) images have been superimposed. *Left*: GFP transgenic embryo. R, retina; B, brain. *Right*: XR11 transgenic embryo. Dorsal is at the top. ***B***. Mean numbers of TUNEL-positive nuclei in sections at stages 28, 33/34, 37/38 and 41. Error bars indicate standard error of the mean. Means were obtained by examining between 14 and 18 embryos for each category. A minimum of 4 and a maximum of 14 sections were examined for each embryo. Pooled data from multiple experiments.

Over-expression of XR11 in nuclear transplant embryos was achieved by utilizing a plasmid containing colinear CMVXR11 and CARGFP genes. CMV is a strong constitutive promoter in *Xenopus*, whereas XCAR promotes gene expression in muscle and cardiac tissue. Thus, XR11 should be expressed universally, whereas expression of the colinear GFP reporter gene in muscle and heart was used to select XR11 transgenics. To determine whether the over-expression of XR11 protected embryonic cells from apoptosis, we compared the incidence of TUNEL-positive nuclei between XR11 and control transgenic whole-mount and sectioned tailbud-stage embryos. Yeo and Gautier [39] demonstrated that the TUNEL assay is effective and specific for detecting apoptosis in *Xenopus *embryos. Examination of control whole-mount tailbud-stage embryos (Fig. 1A, C) showed that darkly labelled apoptotic nuclei are particularly prevalent in the heads, whereas XR11 transgenics (Fig. 1B, D) exhibited little or no detectable apoptosis. Examination of cross-sections (Fig. 2A) revealed that TUNEL-positive nuclei were most prevalent in the retinas and brains, whereas XR11 transgenic embryos lack TUNEL-positive nuclei. Counts of total TUNEL-positive nuclei in sections were made at stages 28, 33/34, 37 and 41 (Fig. 2B). In GFP controls, TUNEL-positive cells peaked at stage 33/34, whereas XR11 overexpressing embryos had nominal numbers of TUNEL-positive nuclei at all stages. For example, over-expression of XR11 at stage 33/34 reduced apoptosis by 95%. Student's *t*-tests indicate that the differences in the numbers of TUNEL-positive nuclei between the XR11 and GFP embryos are highly significant (p = 0.001 for stage 41 and 0.001 > p > 0.000 for stages 28, 33/34 and 37).

### Over-expression of XR11 did not impede apoptosis that occurs during normal or thyroid hormone-induced metamorphosis

**Figure 3:**
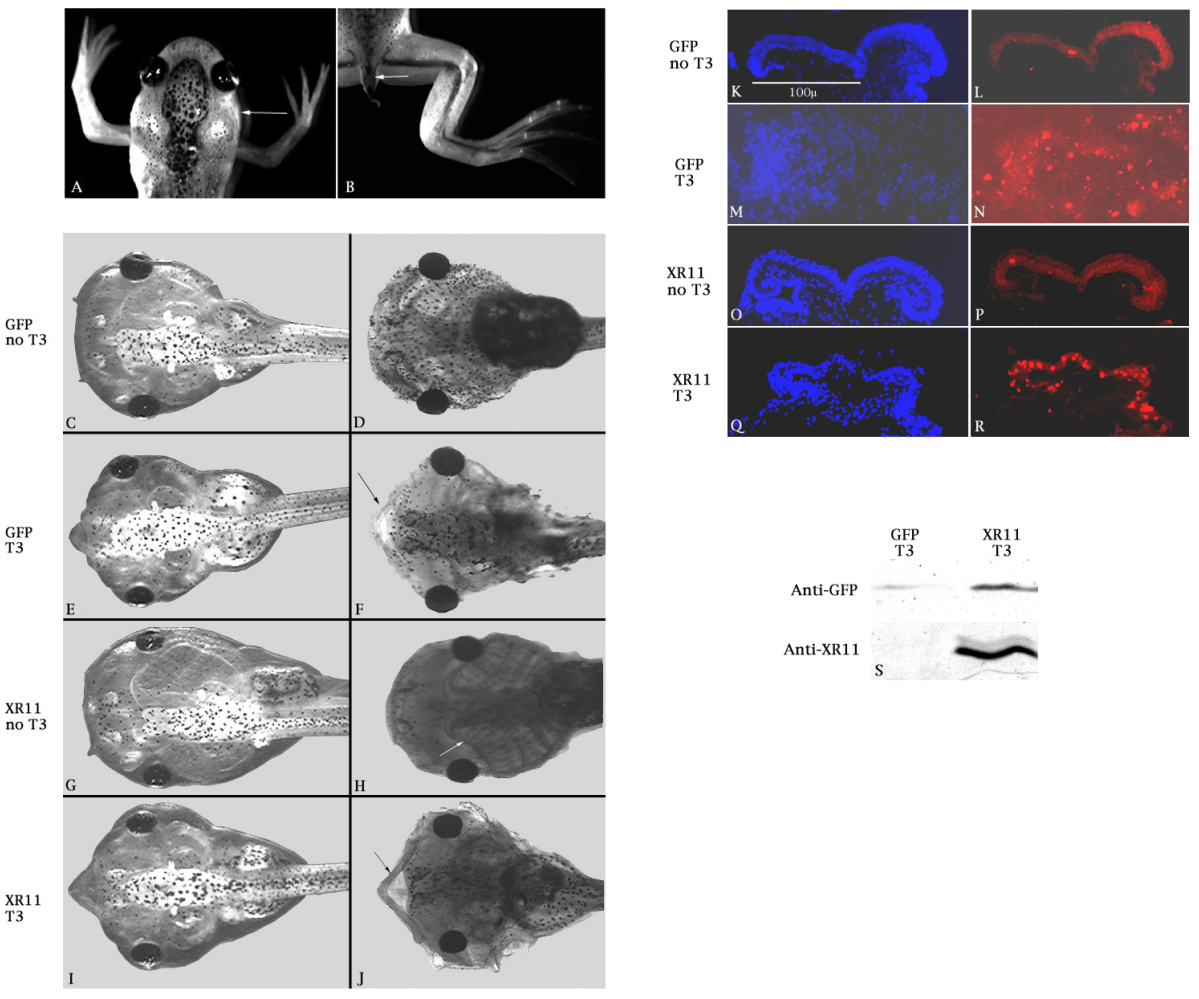
**Metamorphosis of XR11 transgenic tadpoles and responses to exogenous thyroid hormone (T_3_)**. ***A*, *B***. An XR11 transgenic froglet undergoing metamorphosis. ***C-J***. Responses of one-week old (approximately stage 45) transgenic tadpoles to thyroid hormone (T_3_). XR11 transgenic tadpoles (**G-J**;) display the same gross responses to 5 days exposure to as do GFP transgenics (***C-F***). ***C***, ***E, G and I ***are live tadpoles, whereas ***D***, ***F***, ***H ***and ***J ***are fixed tadpoles stained with Alcian blue to reveal the skeleton and the gill apparatus. ***K-R***. The apoptotic response of one-week old transgenic tadpoles (approximately stage 45) to T_3 _was assessed in cross-sections through the velum. DAPI staining (***K, M, O ***and ***Q***) reveals nuclei, whereas the TUNEL assay (***L, N, P ***and ***R***) distinguishes apoptotic nuclei. The velum of the GFP transgenic tadpole (**M, N**) has begun dissociating in response to T_3 _treatment. ***S***. Demonstration of XR11 over-expression in tadpoles by immunoprecipitation. This experiment was repeated once.

If pro-apoptotic factors facilitate apoptosis during metamorphosis of tadpoles into frogs, we would expect over-expression of the anti-apoptotic XR11 to impede metamorphosis. However, XR11 transgenic tadpoles developed as apparently normal larvae that underwent metamorphosis, losing their transient larval structures, such as gills and tails (Fig. 3A, B). The loss of gills is evident by the slimming of the body profile in the pharyngeal region (arrow in *A*). The remnant of the tail is indicated by the arrow in *B*. Loss of these structures occurs via apoptosis during metamorphosis in response to thyroid hormone [42].

To address more specifically whether apoptosis can be triggered by thyroid hormone in XR11 transgenics, we used the metamorphosis induction assay described by Tata [43] and Huang *et al. *[44]. One-week tadpoles were exposed to triiodothyronine (T_3_) for five days. As shown in Figure 3C–J, tadpoles transgenic for XR11 displayed the same gross responses to thyroid hormone, including gill resorption, as shown by the GFP transgenics with no discernable delay. The gill apparatus, which is visible in the absence of T_3 _(arrow in *H*), regresses after T_3 _treatment in both cases. Meckel's cartilage (the lower jaw) shows a dramatic beak-like reshaping (arrows in *F *and *J*).

To determine whether apoptosis was occurring equally in the GFP and XR11 transgenic tadpoles, we focused on the ventral velum, a portion of the larval filter feeding apparatus that has a chevron shape in cross section before it dissociates through apoptosis during metamorphosis [45]. As shown in Figure 3K–R, TUNEL-positive nuclei, which are rare in the velums in the absence of T_3_, were prevalent in the velums of both T_3_-treated GFP and XR11 transgenic tadpoles. These results indicate that apoptosis that occurred in response to thyroid hormone was unaffected by over-expression of XR11. One possible explanation for the extensive apoptosis in the XR11 transgenic tadpoles is that the XR11 protein was no longer over-expressed. However, as shown in Figure 3S, immunoblotting with an antibody to XR11 confirmed that the 22 kDa XR11 protein was over-expressed in the transgenic tadpoles. Proteins were extracted from individual tadpoles at approximately stage 45, separated by electrophoresis, blotted and probed as described in Methods. These tadpoles had been treated with triiodothyronine (T_3_). Similar results were obtained with samples from untreated tadpoles (data not shown). The variation in the GFP signal reflected the intensity of GFP fluorescence in the live tadpoles, presumably reflecting differences in the number of integrated copies of GFP. The 22 kDa XR11 signal is considerably stronger in the XR11 transgenic sample than in the GFP transgenic sample, reflecting XR11 transgene expression.

### XR11 transgenic frogs are fertile and transmit the transgene

The failure of XR11 over-expression to prevent metamorphosis enabled us to rear the resultant frogs to sexual maturity. We induced ovulation in adult females and used the sperm from adult males to obtain second-generation embryos overexpressing XR11. The results of representative crosses are shown in Table 1. Individual parents were designated by number (i.e., XR11-01, XR11-02). Crossing the XR11-01 male with a wild-type female gave a 1:1 ratio of transgene positive to transgene negative progeny, suggesting that his genome had integrated the transgene at a single site. Crossing him with the XR11-01 female gave a 3:1 ratio, a result consistent with a single transgene integration site. However, crossing him with the XR11-02 female yielded a 7:1 ratio, suggesting that this female had two transgene integration sites.

**Table 1:** Representative Crosses Illustrating Inheritance of the XR11 Transgene

**Cross**	**GFP positive**	**GFP negative**	**Ratio (χ^2^)**
XR11-01 male × WT-01 female	197	199	1:1 (p < 0.9)
XR11-01 male × XR11-01 female	319	90	3:1 (p < 0.1)
XR11-01 male × XR11-02 female	321	46	7:1 (p < 0.9)

### Over-expression of XR11 curtailed apoptosis induced by irradiation

**Figure 4:**
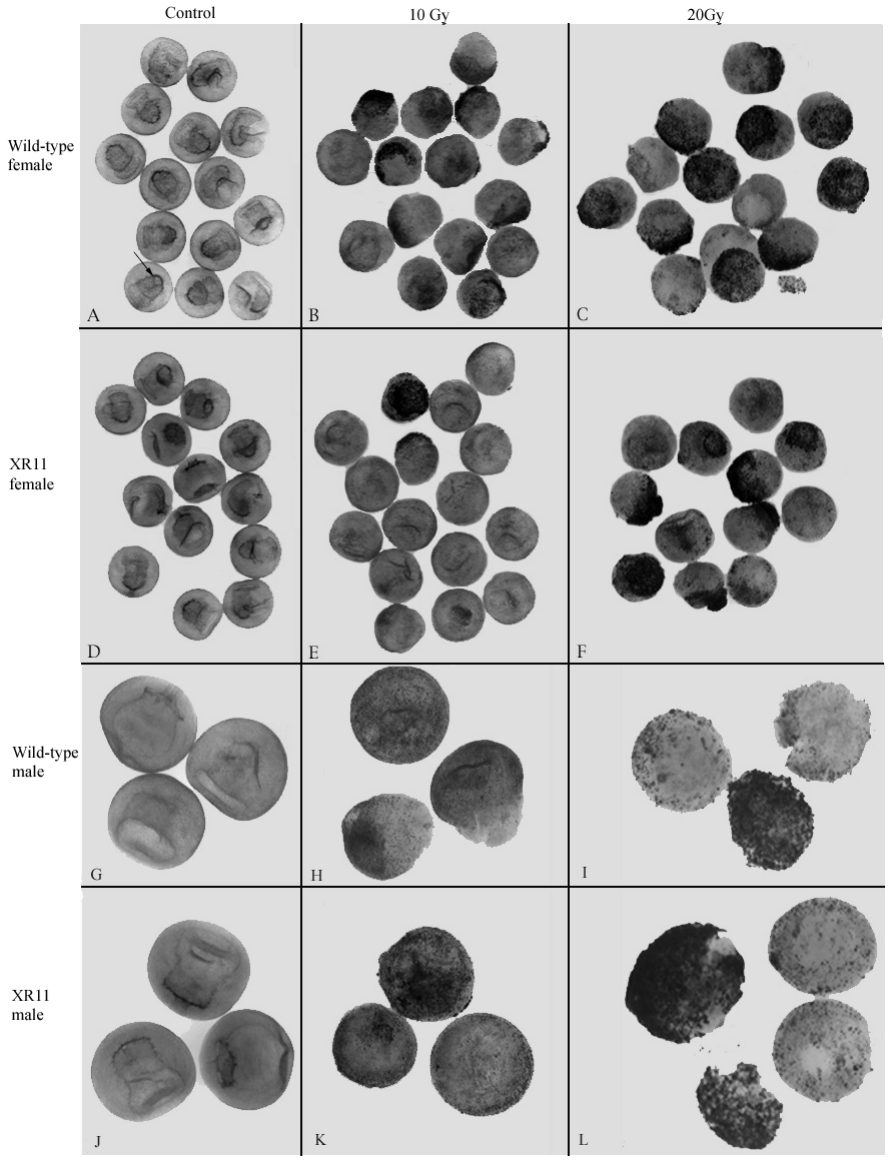
**Maternal and paternal expression of the XR11 transgene**. ***A-F***. Maternal expression of the XR11 transgene reduces the extent of apoptosis in embryos exposed to γ-radiation at stage 6–6.5 and examined 12 hours after irradiation by whole-mount TUNEL assays. ***A-C***, Wild-type embryos. ***A***, Representative control embryos. Arrow indicates the blastopore lip. No evidence of apoptosis is apparent. Embryos that had been exposed to 10 Gy are shown in ***B***. More extensive damage is seen after exposure to 20 Gy (***C***). ***D-F***, TUNEL assays of progeny of fertilization of XR11 eggs by wild-type sperm after exposure to either 10 Gy (***E***) or 20 Gy (***F***). This experiment was conducted three times using eggs from four XR11 females. ***G-L***. Paternal expression of the XR11 transgene does not protect embryos from apoptosis induced by γ-radiation before the MBT. Representative whole-mount TUNEL assays comparing the effects of γ-radiation at stage 6–6.5 on wild-type embryos (***G-I***) and progeny of fertilization of wild-type eggs by XR11 sperm (***J-L***). Embryos were fixed for TUNEL assay 12 hours after irradiation. ***G***, ***J***. Controls. ***H, K***. 10 Gy. ***I, L***. 20 Gy. This experiment was conducted twice using sperm from two XR11 males.

The ability to produce second-generation transgenics gave us the opportunity to compare the effects of maternal and zygotic expression of the XR11 transgene on susceptibility of embryos to γ-radiation-induced apoptosis. Consistent with the literature [15], wild-type embryos subjected to -radiation before the MBT did not undergo apoptosis before the MBT (data not shown). The appearance of TUNEL-positive nuclei was delayed until after the MBT and was radiation dosage-dependent (Fig. 4A–F). After exposure to 10 Gy (Fig. 4B) blastopores were not evident, and large sectors containing TUNEL-positive nuclei were seen. More extensive damage was seen after exposure to 20 Gy (Fig. 4C). TUNEL-positive nuclei were pervasive. Dissociation of the embryos was so extensive that the embryos often fragmented during the TUNEL procedure.

In contrast to the results with wild-type embryos, embryos produced by fertilization of eggs from XR11 females with wild-type sperm showed evidence of partial protection from γ-radiation (Fig. 4E, F). Reduced damage from radiation was always observed after exposure to 10 Gy, but protection from 20 Gy was variable from experiment to experiment, suggesting that overexpression of XR11 was not always sufficient to overcome the effects of 20 Gy.

To investigate whether protection from radiation-induced damage was due to maternal or zygotic expression of the transgene, we irradiated embryos derived from fertilizing eggs of wild-type females with sperm from XR11 males and compared them to wild-type embryos. If zygotic expression of the transgene could partially overcome the effects of pre-MBT γ-radiation, we would have expected to see a subset of embryos with few or no TUNEL-positive nuclei that resembled embryos derived from eggs of XR11 females. Instead, all embryos resembled irradiated wild-type embryos (compare Fig. 4J–L to Fig. 4G–I). Extensive TUNEL-positive nuclei are seen in both groups at both doses. Furthermore, the embryos exposed to 20 Gy fragmented during the TUNEL procedure. These results are consistent with a requirement for a maternal source of radiation protection at these early stages.

**Figure 5:**
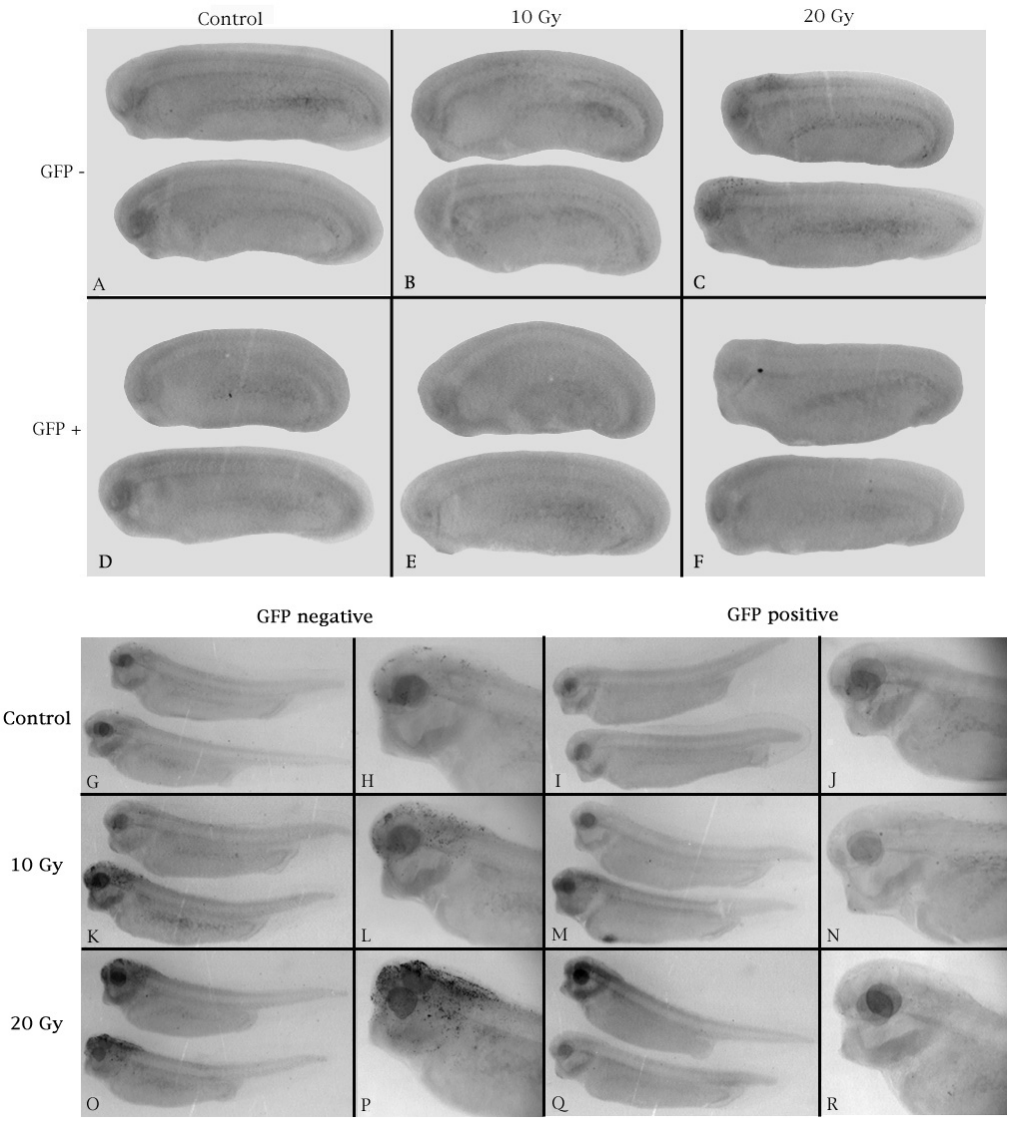
**Representative TUNEL assays showing (*A-F*) the sustained radiation tolerance of early post-MBT embryos and (*G-R*) the effects of zygotic transgene expression in late post-MBT embryos derived from eggs of XR11 females**. (***A-F***). Embryos were irradiated at stage 11.5 and fixed for TUNEL assay 12 hours later. ***A-C***. GFP-negative embryos (lacking the XR11 transgene). ***A***, Control. ***B***, 10 Gy. ***C***, 20 Gy. ***D-F***. GFP-positive embryos (containing the XR11 transgene). ***D***, Control. ***E***, 10 Gy. ***F***, 20 Gy. This experiment was conducted four times using eggs from six XR11 females. ***G-R***. Embryos were irradiated in the mid-30 stages and fixed for TUNEL assay 12 hours later. ***G, H, K, L, O, P***. GFP-negative embryos (lacking the XR11 transgene). ***G, H***. Control. Small numbers of TUNEL-positive nuclei are evidence of spontaneous developmental apoptosis. ***K, L***. 10 Gy. ***O, P***. 20 Gy. ***I, J, M, N, Q, R***. GFP-positive embryos (containing the XR11 transgene). ***I, J***. Control. ***M, N***. 10 Gy. The ventral pigmentation in ***M ***is not due to TUNEL-positive nuclei. ***Q, R***. 20 Gy. This experiment was conducted once using eggs from two XR11 females.

According to the literature, embryos irradiated after the MBT do not undergo apoptosis [17,19,20]. Indeed, embryos that were irradiated after the MBT (stage 11.5) and examined 12 hours later did not show outward signs of radiation damage (data not shown). However, TUNEL assays showed that irradiation enhanced apoptosis in wild-type embryos, predominantly in the brain, eyes, pharynx and tail bud. Hence, we sought to determine whether the maternal effect of the XR11 transgene was sustained after the MBT and whether zygotic expression of the transgene could also confer radiation tolerance. To do this, we segregated the XR11 transgenic embryos of over-expressing XR11 females from their non-transgenic siblings based upon their expression of the GFP reporter gene under control of the cardiac actin promoter. The GFP negative (GFP-) embryos had maternal expression of the XR11 transgene but no zygotic expression, whereas the GFP positive (GFP+) embryos had both maternal and zygotic XR11 transgene expression. Results show that maternal expression of the XR11 transgene protected embryos irradiated at stage 11.5 even if they did not express zygotic XR11. As shown in Figure 5A–F, neither the GFP- nor the GFP+ embryos had extensive apoptosis after irradiation, although some GFP- embryos demonstrated some apoptosis in the hindbrain region, but not in the tail tip (see Fig. 5C). When embryos were irradiated during the mid-30 stages (late tail bud) and examined 12 hours later, the protection afforded by maternal expression of XR11 had diminished significantly in the head and pharynx, although the tail tips were still protected (Fig. 5K, L, O and 5P). However, zygotic expression of XR11 was very effective in conferring global protection from apoptosis (Fig. 5M, N, Q and 5R).

**Figure 6:**
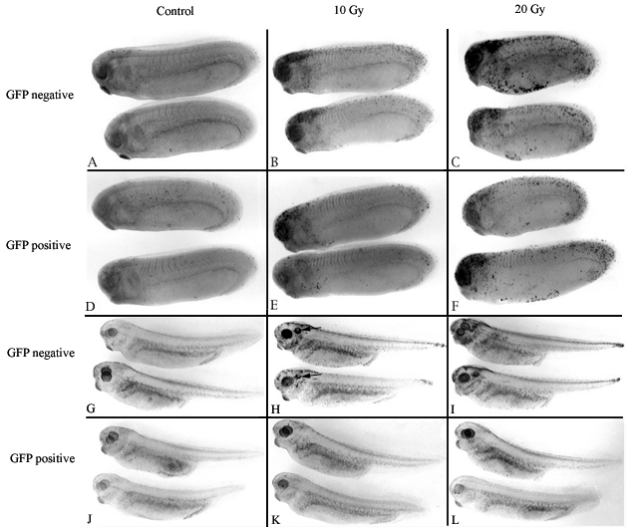
**Representative TUNEL assays showing the effects of zygotic XR11 expression on radiation tolerance of (*A-F*) early post-MBT embryos and (*G-L*) late post-MBT embryos derived from eggs of wild-type females**. ***A-F***. Embryos were irradiated at stage 11.5 and fixed for TUNEL assay 12 hours later. ***A-C***. GFP-negative embryos. ***A***. Control. ***B***. 10 Gy. ***C***. 20 Gy. ***D-F***. GFP-positive embryos (containing the XR11 transgene). ***D***. Control. ***E***, 10 Gy. ***F***. 20 Gy. This experiment was conducted three times using sperm from five XR11 males. ***G-L***. Embryos were irradiated in the mid-30 stages and fixed for TUNEL assay 12 hours later. ***G-I***. GFP-negative embryos. **G**. Control. ***H***. 10 Gy. ***I***. 20 Gy. ***J-L***. GFP-positive embryos (containing the XR11 transgene). ***J***. Control. ***K***. 10 Gy. ***L***. 20 Gy. This experiment was conducted once using sperm from two XR11 males. The experiment could not be repeated due to the unavailability of additional XR11 males.

To examine the effects of zygotic XR11 transgene expression in the absence of maternal expression, we irradiated stage 11.5 embryos derived from fertilizing eggs from wild-type females with sperm from XR11 males. Progeny were scored 12 hours later for GFP expression directed by the cardiac actin promoter to confirm the presence of the transgene. As shown in Figure 6A–F, there was extensive apoptosis in both the heads, pharynges and tail tips of irradiated neurula-stage embryos and no discernable difference in the levels of apoptosis observed between the GFP-positive and GFP-negative embryos. Embryos that received 20 Gy radiation (Figs. 6C, F) are stunted with blunt heads, indicative of extensive developmental defects. These results suggest that zygotic expression of the transgene had little effect in preventing apoptosis at this stage. However, when sibling embryos were irradiated in the mid-30 stages and examined 12 hours later, the transgene conferred global protection from apoptosis (Fig. 6G–L). Note particularly the extensive apoptosis that is evident in the otic capsules (arrows in Fig. 6H) of irradiated embryos lacking the transgene (Figs. 6H, I).

The TUNEL data presented above indicate that: (1) maternal expression of the XR11 transgene reduces the amount of apoptosis induced by γ-radiation during pre-MBT and early post-MBT stages, (2) protection from apoptosis by maternal transgene expression is sustained in the tail tip after tissues in the head become susceptible to apoptosis (irradiation in the mid-30 stages) and (3) radiation tolerance is acquired gradually after the MBT and is sufficient to provide complete protection from apoptosis to embryos irradiated in the mid-30 stages of development.

**Figure 7:**
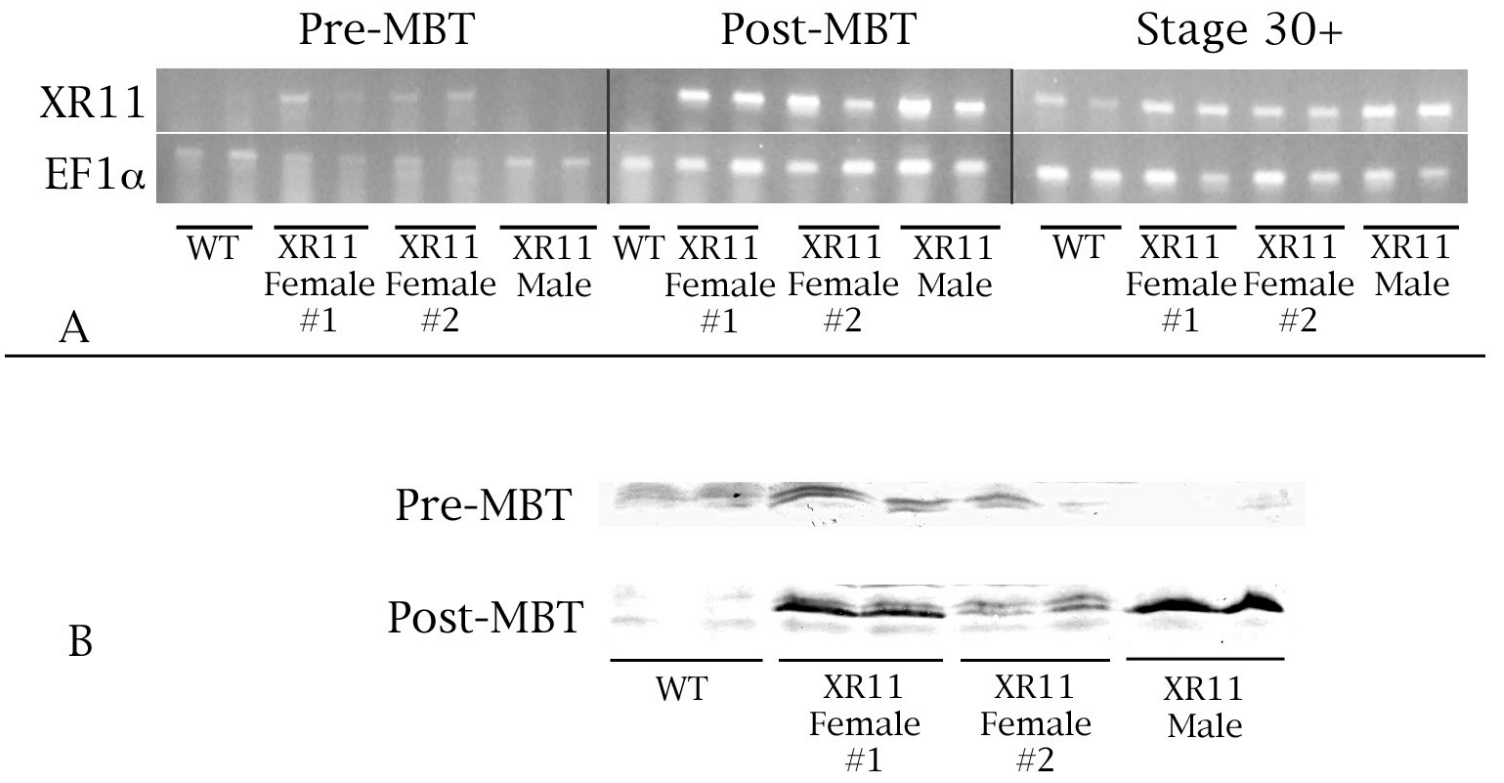
**Maternal and zygotic XR11 RNA expression and protein levels**. **A**. Over-expression of XR11 at the RNA level. RT-PCR of the constitutively expressed elongation factor 1-alpha (EF1α) was conducted in parallel as a control. This experiment was conducted once. **B**. XR11 protein levels in pre- and post-MBT embryos were demonstrated by Western blot analysis. Because the XR11 protein is membrane-bound, it is difficult to separate it from yolk, which is abundant in embryos. The presence of yolk during electrophoresis results in wavy bands in the gel. This experiment was repeated once.

To correlate transgene expression with repression of radiation-induced apoptosis, we monitored XR11 RNA expression and protein levels (Fig. 7). Expression of XR11 at the RNA level in embryos was monitored by RT-PCR with primers designed against the XR11 sequence. RT-PCR was performed on first strand cDNA synthesized from RNA isolated from pre-MBT (stage 7) and post MBT (stage 10.5 and mid-30 stages) wild-type embryos and embryos derived from fertilizing eggs of two different XR11 females with wild-type sperm and the fertilization of wild-type eggs with XR11 sperm. As shown in Figure 7A, we observed maternal XR11 transcripts in pre-MBT embryos derived from transgenic females. On the contrary, there was no evidence for expression of the XR11 transgene in embryos derived from wild-type eggs fertilized by XR11 sperm. After the MBT, XR11 mRNA derived from both maternal and paternal transgenes was evident. Immunoblots (Fig. 7B) indicated the presence of maternally-derived XR11 protein and the accumulation of XR11 protein from either maternal or paternal transgene expression after the MBT.

## Discussion

The application of technology to modify *Xenopus *genetically has created opportunities to conduct functional analyses that are not readily achieved by other means. In this study, we have used XR11 transgenics to examine the role of Bcl-x_L _in suppressing apoptosis during embryonic development, thyroid-hormone-induced metamorphosis and in response to γ-radiation. Our ability to raise XR11 transgenic frogs to maturity has also given us the opportunity to distinguish between maternal and zygotic contributions of Bcl-x_L _to radiation protection.

This study supports the growing evidence in the literature that Bcl-x_L _is sufficient to promote neuronal survival in mice and chicken embryos [32-35]. Our experiments showed that the XR11 transgene could eliminate most apoptosis in the developing *Xenopus *nervous system, including the brain and eyes. This result is consistent with a model in which the execution of developmental apoptosis is dependent upon a balance between pro-and anti-apoptotic factors with Bcl-x_L _being one of the anti-apoptotic factors. In spite of the abatement of developmental apoptosis in XR11 transgenic embryos, there were no apparent gross developmental consequences; the embryos developed into phenotypically normal tadpoles. This result suggests that developmental apoptosis is not essential for development of vital organ systems, notably the central nervous system, and that embryos can adapt to an overabundance of neurons. However, detailed morphometric studies would be necessary to confirm this conclusion. Yeo and Gautier [39] previously showed that injection of human *bcl-2 *mRNA abrogated programmed cell death during early *Xenopus *development, resulting in an expansion of neural tissue and variations in the patterns of expression of a number of genes expressed during early neurogenesis. We did not examine gene expression patterns in our embryos. However, if such alterations did occur, they had no apparent functional consequences.

Contrary to expectation, metamorphosis of XR11 transgenic tadpoles was normal, as was the response to exogenous thyroid hormone. If over-expression of Bcl-x_L _had hindered metamorphosis, it would have indicated that thyroid hormone triggers apoptosis by shifting the balance between pro-apoptotic factors and Bcl-x_L_. However, this proved not to be the case, even though we found that Bcl-x_L _was still misexpressed at metamorphosis. One possibility is that other members of the Bcl-2 family counter apoptosis during these later stages. Another possibility for the failure of XR11 transgenesis to prevent apoptosis during metamorphosis is that expression of the transgene was insufficient to shift the balance between pro-and anti-apoptotic factors sufficiently to prevent apoptosis. It is also possible that subtle changes in the timing, rate or cellular selectivity of metamorphosis did occur that we failed to detect. For example, Coen *et al. *[41] demonstrated selective protection of Rohon-Beard neurons in XR11 over-expressing tadpoles during metamorphosis. We did not assess cell-specific neoronal survival during metamorphosis.

Upon reaching sexual maturity, both male and female frogs were fertile. Crosses of transgenic frogs indicated that most had small numbers of transgene integration sites. This result was consistent with a previous transgene transmission study [46]. The ability to produce second generation transgenic XR11 embryos provided us with the opportunity to examine both the maternal and zygotic contributions of Bcl-x_L _to the apoptotic response to γ-radiation. This provided evidence for a significant role for apoptotic factor balance in determining whether cells that had received damaging dosages of radiation during development were eliminated. Other Bcl-2 family members may carry out similar roles in that microinjection of human *Bcl-2 *mRNA into *Xenopus *embryos is effective in inhibiting irradiation-induced apoptosis [16].

Expression of the XR11 transgene had a profound effect on whether cells of irradiated embryos underwent apoptosis. Furthermore, the application of transgenics allowed us to demonstrate that this effect depended upon the source of the over-expressed protein. Over-expression of Bcl-x_L _derived from XR11 females provided partial protection from the normal massive apoptosis that occurs in irradiated pre-MBT wild-type embryos after reaching the MBT. However, if the XR11 gene was derived from XR11 males, no protection was seen. This result demonstrates conclusively that apoptosis triggered by irradiation before the MBT can be restrained by maternal expression of Bcl-x_L _and that zygotic expression of Bcl-x_L _is ineffective in overcoming the effects of prior irradiation.

Irradiation soon after the MBT (at stage 11.5) had much more subtle effects on embryos. In wild-type embryos, apoptosis was enhanced in the brain, eyes, pharynx and tail tip. However, maternal expression of XR11 was sufficient to abrogate apoptosis. Interestingly, zygotic expression of XR11 at this time was not effective in preventing apoptosis even though XR11 protein was abundant at the time of irradiation. The failure of zygotically-derived XR11 to provide protection from apoptosis implies that synthesis of XR11 protein is not sufficient to prevent apoptosis.

Embryos irradiated during the mid-30 stages of development revealed that the maternal effect conferring protection from radiation-induced apoptosis had diminished significantly in the head and pharynx but persisted in the tails. It will be interesting to learn whether the difference between the heads and tails is due to preferential distribution or stability of maternal XR11 protein itself or is due to auxiliary factors that influence XR11 protein function. Bcl-x_L _and other members of the Bcl-2 family are known to be subject to post-translational regulation by such events as phosphorylation, proteolysis, cleavage, protein-protein interactions and subcellular localization [47,48]. Intriguingly, at these later stages, zygotic XR11 protein conferred global radiation protection. Thus, the initial lack of functionality of zygotic XR11 protein that we observed after the MBT had been overcome.

Biologically, the responses to irradiation after the MBT are probably more significant than the universal, catastrophic apoptosis that occurs in embryos irradiated before the MBT. Failure to eliminate cells mutagenized by irradiation would result in retention of cells that may have functional deficiencies and have the potential to become malignant due to genetic damage. Thus, the mechanisms that control apoptosis in response to DNA damage have important adaptive significance. Radiotherapy and a number of chemicals have been shown to damage DNA and induce apoptosis in mammals; this property has been exploited in the clinic to induce apoptosis of cancer cells. Resistance to apoptosis has important implications for the efficacy of chemotherapy and radiotherapy because tumor cells that overexpress either *bcl-2 *or *bcl-x*_*L *_may be resistant to either mode of cancer therapy [37,49-52]. On the other hand, their over-expression in non-tumor cells could enhance the ability of these cells to survive the otherwise lethal effects of radiation and chemotherapy.

Our results indicate that synthesis of Bcl-x_L _is not sufficient to confer radiation protection; rather, it belatedly acquires functionality. Possible explanations for the failure of Bcl-x_L _synthesized after the MBT to protect against apoptosis could include the absence of posttranslational modifications to the protein or the lack of a cooperative factor that is necessary for the protein to function effectively. The presence of this mechanism that confers functionality on Bcl-x_L _protein before the MBT and its absence soon after the MBT may provide an assay system that would facilitate its identification. The discovery of a mechanism that could control whether cells undergo apoptosis after exposure to an apoptogenic stimulus could have important implications for improving the efficacy of chemotherapy and radiotherapy.

## Conclusion

Our results are consistent with a model in which the execution of developmental apoptosis in embryos is dependent upon a balance between pro-and anti-apoptotic factors with Bcl-x_L _being one of the anti-apoptotic factors. The drastic reduction of apoptosis in XR11 transgenic embryos had no apparent functional consequences. Metamorphosis of transgenic tadpoles was apparently normal, as was the response to exogenous thyroid hormone. The ability to produce second-generation transgenic XR11 embryos enabled us to examine both the maternal and zygotic contributions of Bcl-x_L _to the apoptotic response to γ-radiation. Post-MBT apoptosis that was triggered by irradiation before the MBT was restrained by maternal expression of Bcl-x_L_. However, zygotic expression of Bcl-x_L _was ineffective in overcoming the effects of prior irradiation. Maternal expression of XR11 was also sufficient to abrogate apoptosis triggered by post-MBT γ-radiation. However, zygotic expression of XR11 at this time was not effective in preventing apoptosis even though XR11 protein was abundant.

## Methods

### Transgenesis

Adult *Xenopus laevis *were maintained in compliance with the University of Calgary guidelines for animal care. Our transgenesis protocol is based upon the technique described by Sparrow[53], which is a simplification of the procedure described by Kroll and Amaya [40]. The simplified technique eliminates the need to use egg extract and restriction enzyme to facilitate transgenesis. We have found no significant difference in the frequencies of either normal development, transgenesis or viability of embryos, tadpoles or frogs using these two techniques (data not shown). The simplified transgenic reaction involves mixing 2.5 × 10^5 ^nuclei in 2.5 μl of sperm storage buffer, 100 ng linearized DNA in 2.5 μl of water. The components are mixed and left for 10 minutes at room temperature (~20–23°C). At this point, 495 μl of sperm dilution buffer is added, and the suspension is mixed gently and thoroughly before loading into microinjection needles for nuclear transplantation. Nuclear transplant embryos were screened for expression of the transgene by detecting GFP fluorescence using an Olympus SZX9 fluorescent stereomicroscope.

### Tadpole husbandry

Tadpoles were fed Sera Micron , which is a fine granulated tropical fish food. This proved to be an excellent diet for tadpoles, which were maintained on it until metamorphosis. After metamorphosis, froglets were fed progressively larger forms of NASCO Frog Brittle. Tadpoles were either maintained in plastic food containers in which water was changed manually as needed or in continuously circulating Z-MOD tanks (Marine Biotech, Beverly, MA). After metamorphosis, frogs were maintained in Z-MOD tanks until they became large enough to be transferred to larger-sized X-MOD tanks.

### RNA purification

Whole tadpoles were homogenized in 100 μl TRIZOL (Invitrogen), and 400μl of the reagent were added to the homogenate. After incubation for 5 min at room temperature, 100μl of chloroform were added, the mixture was shaken vigorously for 15 seconds, incubated for 2–3 minutes at room temperature and centrifuged at 12,000 × g for 15 minutes at 4°C. 250 μl of isopropanol were added to the supernatant, which was then incubated for 10 minutes at room temperature and centrifuged at 12,000 × g for 10 minutes at 4°C. The supernatant was removed, and 500 μl of 75% ethanol were added to the pellet. After mixing well, the mixture was centrifuged at 7,500 × g for 5 minutes at 4°C. The supernatant was discarded, and the pellet was air-dried. The dried pellet was rehydrated in RNase-free water and stored at -80°C. Any contaminating DNA was removed with DNA-free (Ambion) according to the manufacturer's protocol. RNA concentration was determined by spectrophotometry at 260 nm.

### RT-PCR

Ready-to-Go RT-PCR beads (Amersham Pharmacia) were used according to the manufacturer's instructions for one-step reactions. RT-PCR reactions for XR11 and EF1α were run in tandem. 500 ng total RNA and 10 pmoles XR11 primers or 5 pmoles EF1α primers were used in each 50 ml reaction. The first strand reaction ran for 15 minutes followed by a 5 minute incubation at 95°C The PCR step ran for 35 cycles for the XR11 product or 30 cycles for the EF1α product. Each cycle was 94°C for 1 minute, followed by 55°C for 1 minute and 72°C for 1 minute. The reaction was stopped by reducing the temperature to 4°C. If necessary, the reaction products could be stored at -20°C until analyzed. The PCR products were analyzed by applying 10 μl samples to a 1.5%agarose/TAE gel containing 0.25 μg ethidium bromide (Sigma-Aldrich) in parallel with a 100 bp ladder (Amersham Pharmacia).

EF1α-downstream primer: ACTGCCTTGATGACTCCTAG

EF1α-upstream primer: CAGATTGGTGCTGGATATGC

This primer pair will generate a 270 bp band.

XR11-downstream primer: GGCATTCTTTCCATACAGGC

XR11-upstream primer: CCTTCTACTTCAGAGCGCCC

This primer pair will generate a 400 bp band.

### Immunoblotting

Antiserum to XR11 was prepared by Alpha Diagnostics International, Inc. (San Antonio TX) in rabbit against a synthetic peptide encompassing amino acids 11–30 (KFV SKK LSQ NEA CRK FSN NP). This antiserum was used for immunoblotting at a dilution of 1:1000. Secondary antibody was goat anti-rabbit conjugated to alkaline phosphatase (Jackson ImmunoResearch), which was used at a dilution of 1:1000.

Individual embryos were homogenized so as to correlate immunoblot results with GFP expression levels. Proteins in tissue homogenates were separated using 15% SDS-polyacrylamide gel electrophoresis and blotted onto PVDF. Blots were blocked either overnight at 4°C or for 1.5 hours at room temperature in 5% skim milk powder in TBS containing 0.1% Tween 20. The blots were then incubated with the primary antibody in TBS containing 0.1% Tween 20 for 1 to 2.5 hr at room temperature, washed 1 × 15 min and 3 × 5 min in TBS containing 0.1%Tween 20. The blots were then incubated with the conjugated secondary antibody in TBS containing 0.1% Tween 20 for 1 hr at room temperature, washed as described above, rinsed with TBS, then with staining buffer without stain reagents, followed by staining with NBT/BCIP until developed.

### Embryo whole-mount TUNEL assays

Pigmented embryos were collected at stages 28, 33, 37, and 41 and processed for TUNEL assays as described by Hensey and Gautier [13]. After staining, the embryos were fixed while rocking overnight at room temperature in Bouin's solution (70 ml water, 25 ml 37% formaldehyde, 5 ml glacial acetic acid). Embryos were then washed (four times for 15 minutes each) in TE buffered ethanol. Embryos were then bleached for 1 hour at room temperature in 0.5 × SSC, 5% formamide, 1%H_2_O_2 _in a Petri dish on a piece of aluminum foil on a shaker under a fluorescent lamp, washed twice for 5 min each in methanol and cleared in BB/BA (2:1 benzylbenzoate:benzyl alcohol). Photographs were taken of embryos representative of the extremes of TUNEL-positive nuclei.

### Cryosectioning

Samples were collected at the appropriate stages and fixed overnight at 4°C in MEMFA (0.1 M MOPS pH 7.4, 2 mM EGTA, 1 mM MgSO_4_, 3.7 % formaldehyde). Samples were then embedded in OCT Compound (Tissue Tek Cat #4583). The embedded samples could then either be stored at -80°C or sectioned immediately. 14 μm sections were made using a Microm cryostat with cabin temperature set at -12°C. Sections were stored at -20°C if not processed immediately. Sections were processed using the ApoTagRed *in situ *Apoptosis Detection Kit, catalogue #7165 according to the manufacturer's protocol (Intergen Co., Purchase NY). Sections were visualized using a Zeiss Axioplan 2 microscope with Spot camera and software.

### Induced metamorphosis

3,3',5-Triiodo-L-thyronine (T_3_; Sigma Cat #T2752) was added one week after fertilization to a final concentration of 10 nM to tadpole water and changed daily for the duration of the experiment [44](Huang *et al.*, 1999). Before and during treatment, tadpoles were maintained in 0.1 × MMR (for description, see Kroll and Amaya [40]) in the absence of food. Tadpoles responded uniformly to the hormone.

### Cartilage staining

Cartilage was visualized in tadpoles according to the procedure described by Klymkowsky and Hanken [54]. Tadpoles were anesthetized in 0.02% benzocaine and immersed in 0.02% Alcian blue 8GX in 70% ethanol/30% glacial acetic acid. After staining, tadpoles were passed through an ethanol series (100, 100, 95, 70, 40, 15%) and then into distilled water. They were then washed in two changes of methanol and cleared in BB/BA.

### Irradiation of embryos

Embryos to be irradiated were placed in ~2.5 ml 0.1 × MMR in 35 × 10 mm plastic Petri dishes (approx. 20/dish) and exposed to either 10 Gy or 20 Gy from a Cesium 137 source in a Gammacell 1000 irradiation chamber (MDS Nordion International Inc., Ottawa, ON Canada). After irradiation, embryos were transferred to 60 × 10 mm plastic Petri dishes containing 0.1 × MMR and allowed to recover for 12 hours at room temperature. Unless noted otherwise, each experiment was repeated at least once.

## List of abbreviations

DAPI: 4',6-diamidino-2-phenylindole dihydrochloride

GFP: green fluorescent protein

MBT: Mid-blastula transition

NBT: Nitroblue tetrazolium

PCD: programmed cell death

SSC: sodium chloride/sodium citrate

T_3_: 3,3',5-triiodothyronine

TBS: Tris-buffered saline

TAE: 40 mM Tris-acetate and 1 mM EDTA

TE: 10 mM Tris-HCl and 1 mM EDTA

TUNEL: TdT-mediated dUTP digoxigenin nick end labeling

## Authors' contributions

JJ conducted the transgenesis experiments, the molecular studies, the thyroid hormone experiments, the irradiation experiments and helped to draft the manuscript. RC assisted in perfecting the transgenesis technique and conducted the experiments on developmental apoptosis. MC-S initiated the radiation experiments. SM participated in the design of the study and helped draft the manuscript. LWB conceived of the study and participated in its design and coordination and helped to draft the manuscript. All authors read and approved the final manuscript.

## Supplementary Material

Additional File 1 Figure 2 Click here for file
